# Ugd Is Involved in the Synthesis of Glycans of Glycoprotein and LPS and Is Important for Cellulose Degradation in *Cytophaga hutchinsonii*

**DOI:** 10.3390/microorganisms13020395

**Published:** 2025-02-11

**Authors:** Wenxia Song, Shaoqi Geng, Qingsheng Qi, Xuemei Lu

**Affiliations:** State Key Laboratory of Microbial Technology, Shandong University, Qingdao 266237, China; swxjiangzui@163.com (W.S.); 202232581@mail.sdu.edu.cn (S.G.); qiqingsheng@sdu.edu.cn (Q.Q.)

**Keywords:** *Cytophaga hutchinsonii*, UDP-glucose 6-dehydrogenase, glycosylation, T9SS, lipopolysaccharide, cellulose degradation

## Abstract

*Cytophaga hutchinsonii*, a member of the phylum *Bacteroidetes*, can rapidly degrade crystalline cellulose through direct cell-to-substrate contact. Most of its cellulases are secreted by the Type IX secretion system (T9SS) and anchored to the cell surface. Our previous study proved that the C-terminal domain (CTD) of the T9SS substrate cellulase Cel9A is glycosylated in *C. hutchinsonii*. However, its glycosylation mechanism has remained elusive. In this study, we found that *chu_3394*, which encodes UDP-glucose 6-dehydrogenase (Ugd), was important for the glycosylation of large amounts of periplasmic and outer membrane proteins in *C. hutchinsonii*. The contents of mannose, glucose, galactose, and xylose were detected to be reduced in the glycoproteins of the ∆*ugd* mutant compared to that of wild-type. They might be essential monosaccharides that contribute to the structure and function of glycans attached to proteins in *C. hutchinsonii*. The depletion of mannose, glucose, galactose, and xylose indicates a decrease in glycosylation modifications in the ∆*ugd* mutant strain. Then, we found that the deletion of *ugd* resulted in weakened glycosylation modification of the recombinant green fluorescent protein-tagged CTD of Cel9A. Additionally, the outer-membrane localization of Cel9A was affected in the mutant. Besides this, Ugd was also important for the synthesis of O-antigen of lipopolysaccharide (LPS). Thus, Ugd was involved in the synthesis of glycans in both glycoproteins and LPS in *C. hutchinsonii*. Moreover, the deletion of *ugd* affected the cellulose degradation, cell motility, and stress resistance of *C. hutchinsonii*.

## 1. Introduction

Glycosylation is an important modification that adds carbohydrates to proteins, thereby affecting many important life processes [1]. Research on glycosylation modification systems in bacteria remains relatively limited and is primarily concentrated on pathogenic bacteria. These studies predominantly focus on several species within the phylum *Proteobacteria*, such as *Campylobacter jejuni* [2], *Helicobacter pylori* [3], *Pseudomonas aeruginosa* [4], and *Haemophilus influenzae* [5]. Additionally, attention has been given to multiple species in the phylum *Firmicutes*, including *Listeria monocytogenes* [6], *Clostridium difficile* [7], and *Streptococcus parasanguinis* [8]. Furthermore, research has also covered certain bacteria from the phylum *Bacteroidete*, such as *Bacteroides fragilis* [9] and *Porphyromonas gingivalis* [10]. In cellulose-degrading bacteria, the glycosylation modifications of cellulases in *Trichoderma reesei* are well-characterized. Endoglucanase Cel7A in *T. reesei* exhibits both N-glycosylation and O-glycosylation. These glycan modifications not only enhance correct protein folding and secretion efficiency within cells but also protect the enzyme from proteolytic degradation [11,12]. Recently, our laboratory found that glycosylation also occurs in *Cytophaga hutchinsonii*, which belongs to the phylum *Bacteroidetes* [12,13,14].

For *C. hutchinsonii* to digest cellulose, the cells need to be in direct contact with the cellulose; in this degradation pathway, many outer membrane proteins, including cellulases, are responsible for the adsorption and preliminary degradation of the cellulose [15]. Most of the cellulases in *C. hutchinsonii* are secreted by T9SS and anchored to the cell surface [12,16]. Our previous study has shown that the T9SS in *C. hutchinsonii* is important for cellulose degradation, absorption of ions, and cell gliding [17,18,19]. The conserved C-terminal domains (CTDs) of T9SS substrate proteins are the outer membrane translocation signals that are recognized by the T9SS [20,21,22]. Our recent studies showed that the CTDs of cellulase Cel9A was glycosylated in *C. hutchinsonii* [13,14]. But the glycosylation modification system of *C. hutchinsonii* is currently unknown. To date, only two glycosyltransferases (CHU_3842 and GtrA) have been identified as taking part in the glycosylation pathway in *C. hutchinsonii* [13,14]. The discovery and investigation of additional enzymes involved in glycosylation modification are imperative for a comprehensive understanding of the glycosylation system of *C. hutchinsonii*. In order to identify genes involved in the glycosylation modification system of *C. hutchinsonii*, we attempted to single knock out over 20 genes involved in glycoside or polysaccharide synthesis and metabolism, including UDP-glucose 6-dehydrogenase.

The enzyme UDP-glucose 6-dehydrogenase (Ugd) is found in many organisms and catalyzes the synthesis of UDP-glucuronic acid (UDP-GlcA) from UDP-Glc [23,24]. Several sugar nucleotides, such as UDP-galacturonate, UDP-D-xylose, and UDP-apiose, need UDP-GlcA as a precursor. UDP-GlcA is also the glucuronosyl donor for the synthesis of several structural polysaccharides [25]. In *Porphyromonas gingivalis*, Ugd participated in the synthesis of O-antigen of lipopolysaccharide (LPS) [26]. In *Acinetobacter baumannii* LUH5549, Ugd3 was involved in capsular polysaccharide synthesis [27]. In *Xanthomonas campestris*, Ugd was involved in the precursor synthesis of xanthan gum. In the luminescent bacterium *Vibrio fischeri*, Ugd was involved in biofilm formation [28]. However, to our knowledge, no studies have reported that Ugd participates in protein glycosylation system in bacteria.

In this study, the function of the *chu_3394* gene in *C. hutchinsonii*, which encodes UDP-glucose dehydrogenase, was investigated. Deletion of *ugd* led to extensive phenotypic changes in *C. hutchinsonii* including cellulose degradation capacity, cell motility, and stress resistance. Lectin blot analysis revealed a diminished response of the outer membrane and periplasmic proteins of the ∆*ugd* mutant to Aleuria aurantia lectin (AAL). The glycoproteins from the wild-type and Δ*ugd* mutant strains were enriched and the monosaccharide composition was tested. In the Δ*ugd* mutant, the levels of mannose, glucose, galactose, and xylose were significantly reduced. Then, a recombinant GFP-CTD_Cel9A_ protein was constructed to study the glycosylation of CTD_Cel9A_. The results showed that there was defective glycosylation of GFP-CTD_Cel9A_ in the Δ*ugd* mutant strain. Furthermore, Western blot showed that Cel9A failed to anchor to the outer membrane of *C. hutchinsonii*. Additionally, the O-antigen was undetectable in the Δ*ugd* mutant. These findings suggest that Ugd was involved in both protein glycosylation modification and glycan synthesis of LPS in *C. hutchinsonii*.

## 2. Materials and Methods

### 2.1. Bacterial Strains, Plasmids, and Culture Conditions

The bacterial strains and plasmids used in this study are listed in Table 1. The primers used in this study are listed in Table 2. The PY6 medium, Stanier medium, and Luria–Bertani medium were consistent with the previous description [17]. *C. hutchinsonii* ATCC 33406 was grown at 30 °C with shaking at 160 rpm in PY6 medium. Stanier medium was used for the filter paper degradation assay and colony spreading assay.

### 2.2. Gene Deletion of Ugd

Gene *chu_3394* (Protein accession number in NCBI: ABG60630) was deleted from *C. hutchinsonii* wild type by linear DNA double crossover. The plasmid Sjhc was used to construct the gene-targeting cassette; a map of Sjhc is shown in Supplementary Figure S1. For the deletion of *chu_3394*, homologous arms *BamH*I-3394H1-*Pst*I and *Sac*I-3394H2-*Xba*I were cloned into plasmid Sjhc by using double enzyme digestion and ligation. The gene-targeting cassette was amplified by primers 3394H1F and 3394H1R from plasmid Sjhc-3394. Approximately 1 μg of the gene-targeting cassette PCR product was transformed into 90 μL of wild-type competent cells by electroporation. After incubation for 5–7 days at 30 °C, positive transformants were screened by PCR and sequencing, then the ∆*ugd* mutant was obtained.

### 2.3. Complementation of the Ugd Deletion Mutant

Plasmid pTSK3328 was used for complementation of the deletion of *ugd*. Primers C3394F/C3394R were used to amplify the complementing fragment C*ugd* from *C. hutchinsonii* wild-type strain genome, including the full length of *chu_3394* and a 500 bp region directly upstream, with a total length of 1.8 kbp. Fragment C*ugd* and pTSK3328 were ligated following *Sac*I/*Sal*I double enzyme digestion to construct the complementation plasmid pTSK3328-C3394. Primers EM3328H1F/EM3328H2R were used to amplify a 6-kbp DNA fragment, including the *ugd* homology arm H1 and H2, erythromycin-resistance gene, and complementation fragment C3394, from pTSK3328-C3394 as the template. Approximately 1 μg of the PCR product was transformed into 90 μL of Δ*ugd* mutant competent cells by electroporation after incubation for 5–7 days at 30 °C.

### 2.4. Cellulose Degradation and Cell Molity Assay

Strains were precultured in Stanier medium. Cells were harvested by centrifugation, washed with carbon-free Stanier medium, and adjusted to OD_600_ = 1.0 for inoculation. Equal amounts of wild-type and mutant cells were then spotted onto Stanier agar plates covered with filter paper for a cellulose degradation assay or onto Stanier soft agar plates for a cell motility assay. Then, the plates were incubated at 30 °C for 10 days.

### 2.5. Detection of Endoglucanases by Zymography

The outer membrane and periplasmic proteins were extracted as described by Wang [29]. Secreted proteins were concentrated using a 30 kDa ultrafiltration device (Millipore, Burlington, MA, USA). Samples were separated by using a native gel. Gels were treated as previously reported, with some modifications for the detection of endoglucanases by zymography [30]. Briefly, the gel was treated three to five times with pre-cooled 0.5 M Triton, with shaking at 4 °C for 15 min each time. Then, 2% (*w*/*v*) sodium carboxymethyl cellulose was added to completely immerse the gel, and it was incubated at 60 °C for 30 min. The gel was then stained with 0.5 M Congo red and washed with 1 M NaCl until the bands were clear.

### 2.6. Extraction of Proteins Fractions and Western Blot Analysis

Different component proteins were obtained as described above. The Western blot program was executed as described by Gao et al. [17]. Samples with equal biomass were subjected to 12% SDS-PAGE for separation and subsequently transferred to a PVDF membrane. The lectins used in this study were purchased from Vectors (Shanghai, China). The anti-GFP monoclonal antibody was purchased from ABclonal (Wuhan, China). All reagents were used in accordance with the manufacturer’s instructions.

### 2.7. Analysis of Monosaccharide Components

The monosaccharide composition analysis of *C. hutchinsonii* glycoproteins was conducted using high-performance liquid chromatography (HPLC) with a C18 column (Agilent, 4.6 mm × 250 mm, 2.7 μm). Glycoproteins (10 mg) were hydrolyzed with 2 M trifluoroacetic acid (TFA) at 110 °C for 10 h. The hydrolysate was dried at 60 °C to remove residual TFA and neutralized with 0.3 M NaOH. Subsequently, 10 µL of lactose (Lac) internal standard was added, followed by thorough mixing with 150 µL of 0.5 M 1-phenyl-3-methyl-5-pyrazolone (PMP). The resulting mixture was incubated in a water bath at 70 °C for 30 min. After neutralization with a 0.3 M HCl solution, the mixture was extracted 6–8 times with chloroform to eliminate excess PMP. Following centrifugation at 6000× *g* for 5 min, the upper aqueous layer was filtered through a 0.22 µm filter membrane, and 10 µL was subsequently injected into the HPLC system [31]. The carbohydrate standard mixture, consisting of mannose, glucosamine, rhamnose, glucuronic acid, galactosamine, glucose, galactose, xylose, arabinose, and fucose, was prepared using the same program. The mobile phase comprised 0.1 M KH_2_PO_4_ (pH 6.8) and methyl cyanide. The flow rate of mobile phase was 0.4 mL min^−1^.

### 2.8. Enrichment of Glycoproteins

Under alkaline conditions, boronic acid groups can bind to cis-dihydroxy structures, forming stable five-membered ring complexes. Since most monosaccharides possess a cis-dihydroxy structure, boronic acid affinity chromatography is an effective method for enriching glycoproteins [32]. Phenylboronic acid is employed to enrich glycoproteins from *C. hutchinsonii*. Briefly, *C. hutchinsonii* cells, cultured to the logarithmic phase, were harvested by centrifugation (5000× *g*, 10 min, 4 °C) and washed with 50 mM piperazine-N, N’-bis (2-ethanesulfonic acid) (PIPES) buffer (pH 6.8). The pellet was resuspended in a binding buffer (20 mM Tris-HCl, 50 mM MgCl_2_, pH 8.5) and lysed using high-pressure homogenization at 700 bar for 3 min. Cell debris was eliminated by centrifugation at 10,000× *g* for 30 min, followed by an additional spin at 100,000× *g* for 45 min. The supernatant obtained after the second centrifugation was designated as the cytoplasmic fraction, while the pellet corresponded to the total membrane fraction [3]. To minimize the influence of polysaccharides, the membrane fraction was resuspended in a binding buffer and centrifuged 2–3 times under the same conditions. Both the cytoplasmic and membrane fractions were filtered and sterilized using a 0.22 µm membrane filter, followed by glycoprotein enrichment with phenylboronic acid. The glycoproteins bound to the chromatography medium were eluted with an elution buffer containing 200 mM sorbitol, 100 mM Tris, and 50 mM EDTA at pH 8.5. The protein samples were dialyzed in double-distilled water (ddH2O) for 24 h and subsequently freeze-dried for further analysis.

### 2.9. Protein Expression in Escherichia coli

The *E. coli* strain W3110 carrying plasmid pBAD24-GFP-CTD_Cel9A_ was cultured in LB medium at 37 °C with shaking at 220 rpm. When the optical density of the culture at 600 nm reached 0.6–0.8, 0.2% L-arabinose (Sigma, St. Louis, MO, USA) was added to induce the expression of green fluorescent protein (GFP)-CTD_Cel9A_ (*C*-terminal domain of Cel9A). After incubation at 28 °C with shaking at 200 rpm for 20 h, the cells were collected and ultrasonicated. The supernatant was used as the intracellular soluble protein after concentration using an ultrafiltration tube (10 kDa, Millipore). The recombinant protein GFP-CTD_Cel9A_ was detected using an anti-GFP monoclonal antibody.

### 2.10. Extraction of LPS

LPS was isolated by using hot aqueous phenol as described by Davis and appropriately modified [33]. Silver staining of LPS samples were performed in accordance with the instructions of the Beyotime rapid silver staining kit (catalog no. P0017S, Beyotime Biotechnology, Shanghai, China).

### 2.11. Disk Diffusion Susceptibility Assay

The disk diffusion assay was performed as described by Zhao [34] and appropriately modified. Five milliliters of cell suspension was spread over solid Stanier plates, and then different toxic reagents were seeded on filter papers in the center of the plates. The plates were incubated at 30 °C to determine the inhibition zone.

## 3. Results

### 3.1. Bioinformatic Analysis and Deletion of Ugd

According to the National Center for Biotechnology Information (NCBI; available online: https://www.ncbi.nlm.nih.gov/), *chu_3394* in the *C. hutchinsonii* genome is annotated as encoding a UDP-glucose 6-dehydrogenase (Ugd). To identify the functions of Ugd, *chu_3394* was deleted from *C. hutchinsonii* wild-type strain. The deletion process is shown in Supplementary Figure S2A. Deletion strains were verified by PCR (Supplementary Figure S2B).

### 3.2. Growth of the ∆Ugd Mutant Was Defective in PY6 Medium

**Figure 1:**
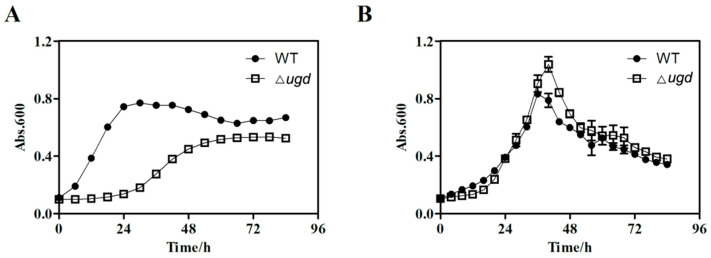
Growth determination of wild-type (WT) *Cytophaga hutchinsonii* ATCC 33406 and the ∆*ugd* mutant. (**A**) Growth curves of cells in PY6 medium. (**B**) Growth curves of cells in Stanier medium. WT—*C. hutchinsonii* wild-type strain; ∆*ugd*—*chu_3394* deletion strain. Growth was determined from absorbance values at 600 nm. Three biological replicates were set, and the mean values and SDs are shown.

To investigate the growth of the Δ*ugd* mutant, growth curves were determined in two different media. The Δ*ugd* mutant showed an obvious growth defect in PY6 medium, with a lag phase of >24 h and slower growth compared with that of the wild-type strain (Figure 1A). Using the maximum absorbance value at 600 nm as an indicator of the maximum biomass of the strains, the maximum biomass of the Δ*ugd* mutant strain is only 65% that of the wild type. These results indicate that there are significant growth differences between the wild type and Δ*ugd* mutant in PY6 medium. However, in Stanier medium, a minimal medium with Stanier mineral salts, the growth of the Δ*ugd* mutant was nearly the same as that of the wild type (Figure 1B). Therefore, Stanier medium was used to study the properties of the Δ*ugd* mutant.

### 3.3. Deletion of Ugd Resulted in Defects in Cellulose Degradation and Cell Motility

**Figure 2:**
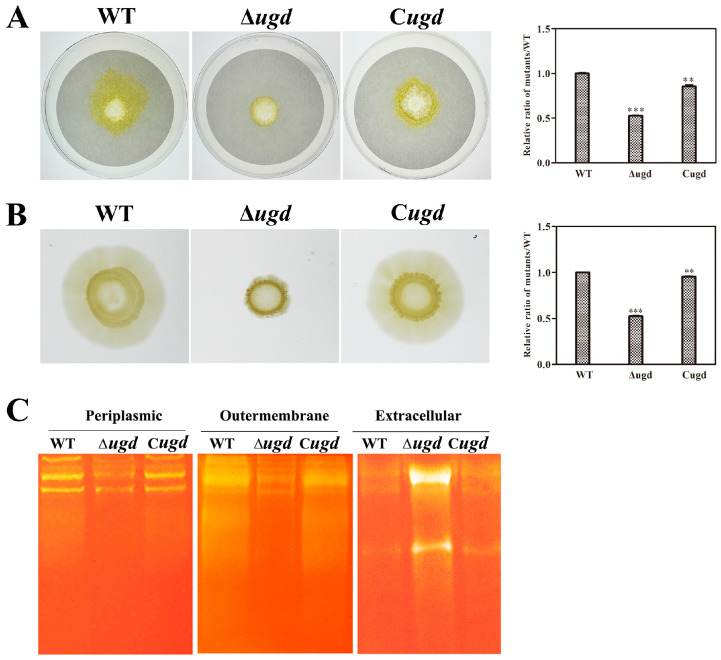
Cellulose degradation ability and colony spreading of the wild-type and Δ*ugd* mutant. (**A**) Filter paper degradation ability. (**B**) Colony spreading ability. (**C**) Detection of endoglucanases by zymography. WT—*C. hutchinsonii* wild-type strain; ∆*ugd—chu_3394* deletion strain. C*ugd*—complementation of Δ*ugd* mutant. All assays were performed in triplicate using three independent transformants, which yielded consistent results, and a representative result is presented. Significance is reported as ** *p* < 0.01, *** *p* < 0.001.

The Δ*ugd* mutant exhibited similar growth to the wild type in liquid Stanier medium. Therefore, Stanier medium was selected to evaluate the cellulose degradation ability and cell motility of the Δ*ugd* mutant. The wild type could grow rapidly on plates of Stanier agar covered with filter parer, while the Δ*ugd* mutant was defective in filter paper degradation (Figure 2A). *C. hutchinsonii* can rapidly glide over solid surfaces without flagella or type IV pili [35]. Colony spreading of the wild-type and the Δ*ugd* mutant was determined on Stanier soft-agar plates. The wild-type cells formed spreading colonies on soft agar, while the ∆*ugd* mutant cells had lost the ability to spread on this medium (Figure 2B). According to the Image J 1.46r analysis results, the filter paper degradation zone of the ∆*ugd* mutant is only 0.525 times that of wild type and the colony spreading zone of the ∆*ugd* mutant is only 0.527 times that of wild type. The complemented strain C*ugd* could degrade cellulose and spread similarly to the wild type on soft agar.

The degradation of cellulose by *C. hutchinsonii* requires direct contact between the cells and the substrate. Therefore, the proper localization of cellulase on the cell surface is very important [16]. According to a previous report, the genome of *C. hutchinsonii* is predicted to encode 18 cellulases, of which 12 are substrate proteins of T9SS. They are transported across the outer membrane by T9SS and anchored to the cell surface to exert their function [16]. To explore the locations of cellulases in the wild type and the Δ*ugd* mutant, the endoglucanases in different cellular fractions were detected by zymography. As shown in Figure 2C, the endoglucanases of the wild type were mainly present in the periplasmic and outer membrane fractions. The cellulase detected in the periplasmic fraction of the Δ*ugd* mutant was slightly reduced compared to the wild type, while the cellulase in the Δ*ugd* mutant outer membrane fraction was significantly diminished. In contrast, the amount of endoglucanases in the extracellular fraction was markedly increased. We inferred that in the Δ*ugd* mutant, the cellulases are unable to anchor on the outer membrane and are released to the culture supernatant.

### 3.4. Ugd Was Involved in Protein Glycosylation in C. hutchinsonii

**Figure 3:**
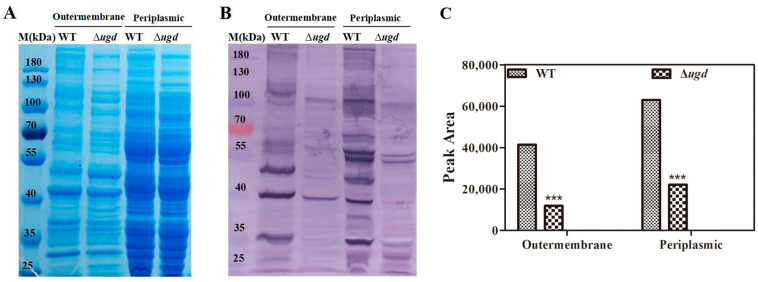
UDP-glucose 6-dehydrogenase (Ugd) is involved in glycosylation in *C. hutchinsonii*. (**A**) SDS-PAGE analysis of outer membrane and periplasmic proteins from the wild type and ∆*ugd* mutant strains, with sample loading normalized to equal biomass. (**B**) Western blot analysis demonstrating that Aleuria aurantia lectin (AAL) recognizes glycoproteins in the wild-type strain, while only a limited number of glycoprotein bands are detected in the ∆*ugd* mutant. (**C**) Quantitative analysis of Western blot band intensity in Figure 3B using Image J 1.46r. Significance reported as *** *p* < 0.001. WT—*C. hutchinsonii* wild-type strain; ∆*ugd*—*chu_3394* deletion strain.

Our previous studies have shown that glycosylation modification is present in *C. hutchinsonii* [13,14]. Different lectins can specifically recognize glycoforms and serve as valuable tools for verifying protein glycosylation modification. We extracted the periplasmic and outer membrane proteins from both wild-type and ∆*ugd* mutant strains and conducted Western blot analysis using five different lectins, including Concanavalin A (ConA), Aleuria Aurantia Lectin (AAL), Sambucus Nigra Lectin (SNL), wheat germ agglutinin (WGA), and soybean agglutinin (SBA). The lectins employed in this study, along with their corresponding recognized structures, are listed in Table S1 [36,37]. We found that AAL can specifically recognize the *C. hutchinsonii* glycoprotein. A substantial number of outer membrane and periplasmic proteins in the wild-type strain were recognized by AAL, indicating that these proteins are glycosylated. However, only a limited number of proteins were detected in the ∆*ugd* mutant (Figure 3B).

### 3.5. The Effect of Ugd Deletion on Monosaccharide Components of Glycoproteins

**Figure 4:**
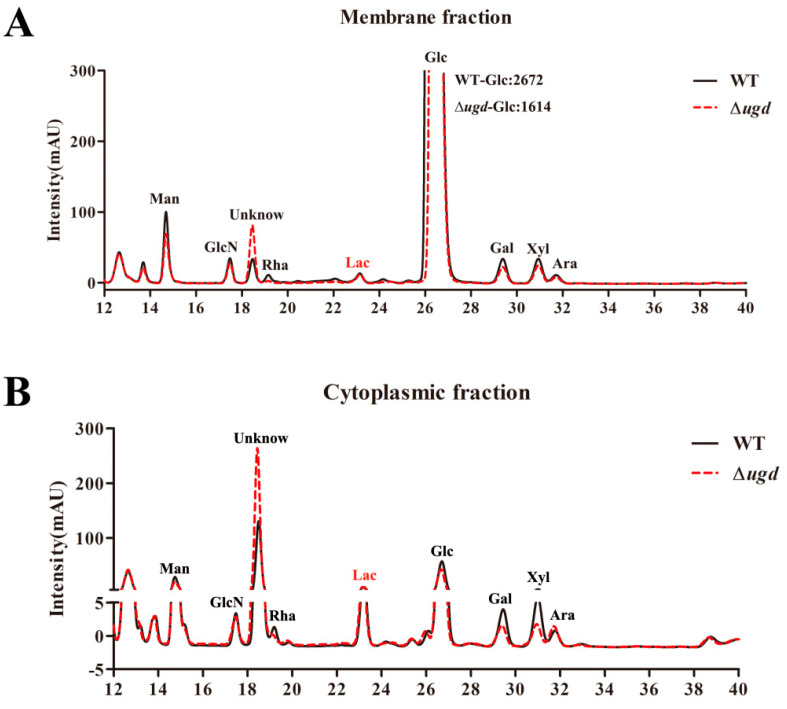
HPLC chromatogram of *C. hutchinsonii* glycoproteins PMP derivatization. (**A**) Analysis of monosaccharides in membrane glycoproteins of wild-type and ∆*ugd* mutant strains by HPLC. (**B**) Analysis of monosaccharides in cytoplasmic glycoproteins of wild-type and ∆*ugd* mutant strains by HPLC. WT—*C. hutchinsonii* wild-type strain; ∆*ugd*—*chu_3394* deletion strain; Man—mannose; GlcN—glucosamine; Rha—rhamnose; Lac—lactose; Glc—glucose; Gal—galactose; Xyl—xylose; Ara—arabinose. Lactose is used as an internal standard. Three independent biological replicates were performed, yielding consistent results. A representative data set is presented.

To investigate the monosaccharide component involved in glycoprotein conjugation in *C. hutchinsonii* and the effect of Ugd on protein glycosylation, we extracted cytoplasmic and membrane proteins from wild-type and ∆*ugd* mutant strains. Glycoproteins were then enriched via boric acid affinity chromatography. The samples underwent PMP derivatization, and the monosaccharide components were analyzed by HPLC. As shown in Figure 4, seven monosaccharides were detected in the glycoproteins from *C. hutchinsonii*, with peak times matching those of the standard sample. An unknown monosaccharide was detected around 18.5 min. The total monosaccharide content in glycoproteins from the membrane components was higher than that from cytoplasmic components. Deletion of the *ugd* gene reduced the levels of mannose, glucose, galactose, and xylose in both the membrane and cytoplasmic glycoproteins compared to the wild-type strain. There was no significant difference in the content of glucosamine and arabinose between the glycoproteins of the wild-type and the ∆*ugd* mutant. Conversely, the content of an unidentified monosaccharide was increased in the ∆*ugd* mutant. These results indicate that Ugd plays an important role in the glycan synthesis of *C. hutchinsonii* glycoproteins.

### 3.6. Ugd Was Involved in the Glycosylation and Location of Cel9A

**Figure 5:**
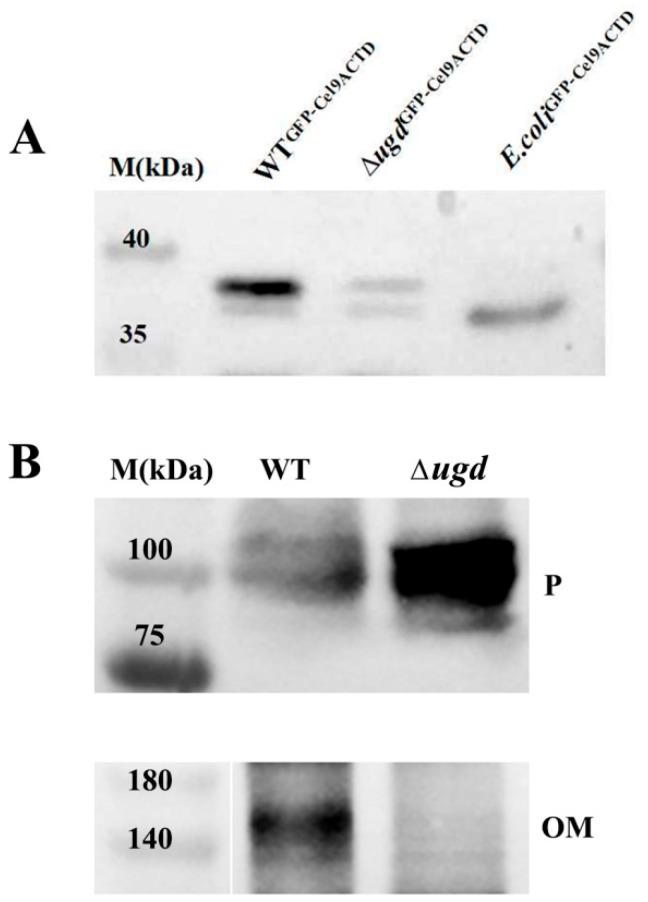
Ugd is involved in the glycosylation and location of Cel9A. (**A**) Detection of periplasmic fraction proteins from WT^GFP-Cel9ACTD^, ∆*ugd*^GFP-Cel9ACTD^, and *E. coli*^GFP-Cel9ACTD^ strains using an anti-GFP antibody. (**B**) Localization and secretion of type IX secretion system (T9SS) substrate protein Cel9A in the wild-type and the ∆*ugd* mutant using an anti-Cel9A antibody. WT—*C. hutchinsonii* wild-type strain; ∆*ugd*—*chu_3394* deletion strain; P—periplasmic proteins; OM—outer membrane proteins.

Cellulase Cel9A (CHU_1336) is a T9SS substrate protein with a CTD in *C. hutchinsonii*. Our previous studies showed that the fusion protein GFP-CTD_Cel9A_ was glycosylated in the periplasm of *C. hutchinsonii* [13]. To assess the impact of Ugd on the glycosylation of GFP-CTD_Cel9A_, we expressed the fusion protein in the ∆*ugd* mutant strain, with GFP-CTD_Cel9A_ expressed in *E. coli* serving as a negative control without glycosylation. As shown in Figure 5A, two GFP-related bands were detected in wild-type *C. hutchinsonii*. According to our previous research [13], the higher-weight band was the target protein modified by glycosylation. When GFP-CTD_Cel9A_ was expressed in the ∆*ugd C. hutchinsonii* mutant, the glycosylated GFP-related band in the periplasm was decreased in intensity compared with that in the wild-type strain (Figure 5A). Based on the above results, we suggested that the Ugd contributes to glycosylation in Cel9A.

T9SS substrate proteins are often anchored to the outer membrane after being secreted [38]. To investigate the location of Cel9A, Western blot analysis was performed using a Cel9A-specific antibody. Cel9A was detected in the outer membrane of wild-type *C. hutchinsonii*, but was absent from the outer membrane of the ∆*ugd* mutant (Figure 5B). Instead, Cel9A accumulated in the periplasmic space of the ∆*ugd* mutant and was also present in the culture supernatant (Figure 5B). Interestingly, the molecular weight of Cel9A found in the periplasm and culture supernatant was approximately 100 kDa, matching the theoretical value based on its amino acid sequence (105 kDa). However, mature Cel9A in the outer membrane was detected, with a molecular weight of >130 kDa, suggesting it may have undergone an additional unknown modification after being transported to the cell surface. Further investigation is required to resolve this issue.

### 3.7. Ugd Is Important for the Synthesis of LPS

**Figure 6:**
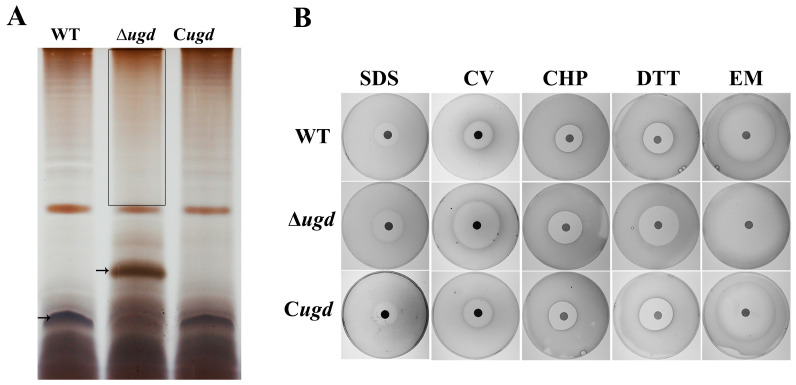
Lipopolysaccharide (LPS) and disk diffusion susceptibility assays for the wild-type, ∆*ugd*, and C*ugd* (*chu_3394* complementation) strains of *C. hutchinsonii*. (**A**) Silver staining analysis of wild-type and ∆*ugd* LPS extracts. The part in the box is the O-antigen repeat unit of LPS; the arrow indicates the LPS core. (**B**) Cell sensitivity to toxic reagents of the wild-type, ∆*ugd*, and C*ugd* (*chu_3394* complementation) strains. SDS–sodium dodecyl sulfate; CV–crystal violet; CHP—cumene hydroperoxide; DTT—dithiothreitol; EM—erythromycin. Three biological replicates were set, and a representative result is presented.

LPS was extracted from *C. hutchinsonii* wild type, Δ*ugd* mutant, and complemented strain C*ugd* by using hot aqueous phenol. The LPS samples were separated by Tricine SDS-PAGE and then visualized with silver stain. As shown in Figure 6A, the integrity of the LPS was disrupted by deletion of *ugd*. In Figure 6A, the part in the box is the O-antigen repeat unit of LPS, and the arrow indicates the LPS core. The O-antigen was missing in the Δ*ugd* mutant, and the lipid A core was heterogeneous in the mutant, with an increased molecular weight.

LPS is an important outer membrane component of Gram-negative bacteria, serving as a protective barrier against toxic agents. Truncation of LPS, particularly the O-antigen, results in a loss of this barrier, making the membrane more susceptible to external damage [39]. Therefore, the tolerance of the wild-type and the ∆*ugd* mutant to various reagents was determined on Stanier medium containing 0.5% (*w*/*v*) agar and 0.2% (*w*/*v*) glucose. The test reagents included 20% (*w*/*v*) sodium dodecyl sulfate, 1% (*w*/*v*) crystal violet, 0.5 M cumene hydroperoxide, 2 M dithiothreitol, and 100 μg/mL erythromycin. As shown in Figure 6B, the Δ*ugd* mutant cells were more sensitive to these reagents. Based on the statistics of the diameter of the inhibition zones and P-value analysis, it can be observed that the Δ*ugd* mutant cells are particularly sensitive to crystal violet, dithiothreitol, and erythromycin. The inhibition zones produced by the complementation strain C*ugd* showed no significant difference compared to the wild type (Supplementary Figure S5).

## 4. Discussion

T9SS substrate proteins were reported to contribute to cellulose degradation, cell motility, toxicity, and digestion of macromolecular substances [40,41,42]. Many cellulases in *C. hutchinsonii* are secreted to the cell surface through the T9SS. Previous studies in our laboratory have found that some T9SS substrate proteins in *C. hutchinsonii* are glycosylated in the periplasmic space, and glycosylation has effects on the secretion and localization of substrate proteins [14]. However, understanding of the glycosylation modification system in *C. hutchinsonii* remains very limited. Until now, only glycosyltransferase CHU_3842 and GtrA have been reported to be involved in the glycosylation process in *C. hutchinsonii* [13,14]. Here, we identified another enzyme, UDP-glucose 6-dehydrogenase, which was involved in not only protein glycosylation but also in LPS synthesis of *C. hutchinsonii*. Lectin blotting analysis revealed a significant reduction in glycoproteins recognized by AAL in the outer membrane and periplasmic components of the ∆*ugd* mutant strain compared to the wild-type strain (Figure 3B). The deletion of *ugd* resulted in a reduction in several monosaccharides, including mannose, glucose, galactose, and xylose, in the glycoproteins of *C. hutchinsonii* (Figure 4). AAL specifically binds to fucose residues; however, no peak corresponding to fucose was detected in the HPLC analysis of the glycoforms of these glycoproteins (Figure 4). This discrepancy may be due to the lower sensitivity of HPLC compared to lectin blotting. To our knowledge, no previous report has demonstrated the involvement of Ugd in glycosylation modification in bacteria. But further experiments are needed to demonstrate the involvement of Ugd in the glycosylation modification pathway of *C. hutchinsonii*.

LPS is an important part of the outer membrane of Gram-negative bacteria, which helps to maintain the integrity of the outer membrane [23]. In *C. hutchinsonii*, the ∆*ugd* mutant produced a truncated O-antigen of LPS, which led to migration above the band corresponding to the lipid A core in LPS silver staining result. Meanwhile, the O-antigen repeat units disappeared in the ∆*ugd* mutant (Figure 6A). In Burkholderia, OgcE is a UDP-glucose/galactose epimerase. Lipid A of LPS in a ∆*ogcE* mutant showed an upwardly heterogeneous state [43]. It was concluded that, because of the deletion of *ogcE*, UDP-GalNAc was absent, and the A-core combined with plus Rha-QuiNAc disaccharide, leading to an upshift in the position of lipid A in the LPS profile [43]. In the ∆*ugd* mutant of *C. hutchinsonii*, the reason for the lipid A upshift might be similar to that in the ∆*ogcE* mutant of Burkholderia. LPS are important cell barriers of Gram-negative bacterium. The deletion of *ugd* results in LPS deficiency, which might be a contributing factor to the increased sensitivity of Δ*ugd* cells to toxic reagents (Figure 6B).

*C. hutchinsonii* is capable of rapidly and efficiently degrading cellulose. However, the mechanism of its cellulose degradation has long been unknown. In this study, the deletion of *ugd* resulted in defects in cellulose degradation. A previous study has predicted that the genome of *C. hutchinsonii* encodes 18 cellulases, of which 12 are predicted to be T9SS substrates. These cellulases are transported by the T9SS system and anchored to the cell surface. The degradation of cellulose by *C. hutchinsonii* requires direct contact between the cells and the substrate; therefore, the normal anchoring of cellulase on the cell surface after translocation across the outer membrane is crucial [16]. Cel9A is a family-9 endoglucanase with a conserved CTD, expected to be located on the cell surface [44]. We found that in the ∆*ugd* mutant, the glycosylation modification of Cel9A is weakened (Figure 5A). Moreover, in the ∆*ugd* mutant, Cel9A failed to anchor on the outer membrane, but was accumulated in the periplasm or released into the culture supernatant (Figure 5B). We speculate that the accumulation of T9SS substrate in the periplasm might be caused by defective glycosylation of the substrate. However, it remains to be studied how glycosylation modification of the substrate proteins affects their transportation across the outer membrane via the T9SS.

In summary, the deletion of *ugd* had a broad impact on the phenotypes of *C. hutchinsonii*, including cellulose degradation, cell motility, and stress resistance. Ugd catalyzes the synthesis of UDP-glucuronic acid (UDP-GlcA), which serves as a direct or indirect precursor for various sugar nucleotides. Additionally, UDP-GlcA acts as a glucuronosyl donor in the biosynthesis of several structural polysaccharides. We speculated that Ugd influenced these phenotypes by participating in the synthesis of protein-linked glycans and LPS glycans. There are still many mysteries about the cellulose degradation and the glycosylation system of *C. hutchinsonii*. Our future work will focus on identifying more genes involved in glycosylation to comprehensively elucidate the glycosylation system of *C. hutchinsonii*, which will facilitate a deeper understanding of its efficient and rapid cellulose degradation mechanisms.

## Figures and Tables

**Table 1:** Strains and plasmids used in this study.

Strains, Plasmids	Description ^a^	References or Source
Strains		
*C. hutchinsonii* strains		
ATCC 33406	Wild type	Laboratory preservation
Δ*ugd* strain	Deletion mutant of *chu_3394*	This study
C*ugd* strain	Complementation of Δ*ugd* mutant with pTSK3328-3394	This study
WT^GFP-Cel9ACTD^ strain	WT containing promoter_2708_-signal peptide_2708_-GFP-CTD_Cel9A_	[13]
Δ*ugd*^GFP-Cel9ACTD^ strain	Δ3394 containing promoter_2708_-signal peptide_2708_-GFP-CTD_Cel9A_	This study
*E. coli* strains		
DH5α	Strain used for plasmid replication	Purchased from Tsingke (Beijing, China)
W3110 (DE3)	Strain used for protein expression	Purchased from WEIDI (Shanghai, China)
Plasmids		
Sjhc	Gene deletion template plasmid carrying Cm flanked by two MCS; Ap*^r^* (Cm*^r^*)	This study
Sjhc-3394	Gene deletion template plasmid carrying *chu_3394*-targeting cassette; Ap*^r^* (Cm*^r^*)	This study
pTSK3328-C3394	Plasmid constructed from pTSK3328 used for complementation of Δ*ugd*; Ap*^r^* (Em*^r^*)	This study
pBAD24	Expression vector; Ap*^r^*	Laboratory preservation
pBAD24-GFP-CTD_Cel9A_	Plasmid constructed from pBAD24 for expression of GFP-CTD_Cel9A_; Ap*^r^*	This study

^a^ Cm*^r^* (chloramphenicol resistance) and Em*^r^* (erythromycin resistance) were expressed in *C. hutchinsonii*, and Ap*^r^* (ampicillinresistance) was expressed in *E. coli*.

**Table 2:** Sequences of primers used in this study.

Primers	Sequence ^a^ (5′-3′)	Purpose
3394H1F	CGGGATCCGTAATTTAAGACGGTAAGAAGCACG	Cloning 3394H1
3394H1R	AACTGCAGCCGTTACTAAACCAACGTATCCC	
3394H2F	ACGAGCTCCTGATCGTTACCGAATGGTCTGA	Cloning 3394H2
3394H2R	GCTCTAGAATCTTTGCGGCTTCTGCTACTTT	
3394H1UF	CGGATACCAGTGCCATACAACAA	Deletion validation of *chu_3394*
3394UR	CTGAGGAGCATCTATACCAACCA	
C3394F	GCGTCGACCTACAGGAAATTCCTGTACTTCATCCCATTTC	Cloning C3394
C3394R	ACGAGCTCTTATTCTGCTTTTAGTCCGATGCAGTAGTAATC	
EM3328H1F	TTAGCATGCTCTGTTGAGCAGGTTCTACTGGG	Cloning fragment 3328H1-EM-C3394-3328H2
EM3328H2R	ATAGGATCCTCTATAATTGGCTGACCGACACG	
EM3328UF	AGTAAGCGGTATGTGTGAAATGTGC	Complementation validation of *chu_3394*
EM3328UR	GTTGCCTTCTTTGATTATGCCGTC	
C3394RYZ	GGTTAAGTAGCATTATAATGGAATACGTTG	
CM-R	GTTTTATCCGGCCTTTATTCACATT	Deletion validation of *chu_3394* with primer 3394H1UF
PB-GFPF	CGCCCGGGATGAAAAAAGTTTTACTTTCTTTATCAATGC	Cloning GFP-CTD_Cel9A_ expressed in *Escherichia coli*
PB-GFPR	AGGTCGACTTAGTATTTAATGATATTCATCG	
PB-YZF	CGCAACTCTCTACTGTTTCTCC	Validation GFP-CTD_Cel9A_ expression in *Escherichia coli*
PB-YZR	CAGACCGCTTCTGCGTTCTG	

^a^ The underline indicates the restriction endonuclease cleavage site in the primer.

## Data Availability

The original contributions presented in this study are included in the article. Further inquiries can be directed to the corresponding author.

## Supplementary Materials

The following supporting information can be downloaded at: https://www.mdpi.com/article/10.3390/microorganisms13020395/s1, Figure S1: Map of the plasmid Sjhc; Figure S2: The deletion of *ugd*; Figure S3: HPLC chromatogram of standard monosaccharide PMP derivatization; Figure S4: The outer membrane and periplasmic proteins of wild-type and ∆*ugd* mutant were verified by Western blot and antibody-assisted lectin profiling; Figure S5: The diameters of the inhibition zones for wild type, ∆*ugd*, and C*ugd* strains treated with various toxic agents; Figure S6: Diagram illustrating Ugd’s involvement in the nucleotide sugar biosynthesis pathways of *C. hutchinsonii*. The deletion of Ugd in *C. hutchinsonii* disrupts one of the UDP-GlcA biosynthesis pathways, which in turn affects the synthesis of various nucleotide sugars, including UDP-D-Xyl, UDP-D-Api, and UDP-L-Ara4O; Table S1: The lectins used in this study.
