# Supplementary material for: Ugd Is Involved in the Synthesis of Glycans of Glycoprotein and LPS and Is Important for Cellulose Degradation in Cytophaga hutchinsonii
Source: Microorganisms. 2025 Feb 11;13(2):395. doi: 10.3390/microorganisms13020395 (PMC11858162; doi:10.3390/microorganisms13020395)
Supplement: Supplementary file 1 [file microorganisms-13-00395-s001.zip › microorganisms-3435247-supplementary.pdf]

Supplementary Material

Table S1 The lectins used in this study

Lectin	Specific glycoform
Aleuria Aurantia Lectin (AAL)	Combining fucose linked with N-acetylglucosamine or N-acetylglucosamine
Concanavalin A (ConA)	Combining certain structures in α - mannose and α - glucose residues
Sambucus Nigra Lectin (SNL)	In α-2,6 and to a lesser extent α-2,3 linkages, it preferentially binds to sialic acid attached to the terminal galactose, seemingly not to sialic acid linked to N-acetylgalactosamine
Wheat germ agglutinin (WGA)	Combining N-acetylglucosamine (GlcNAc), it preferentially binds to the dimers and trimers of the sugar. WGA can bind oligosaccharides containing N-acetylglucosamine or chitosan at the end
Soybean Agglutinin (SBA)	Prioritize binding to oligosaccharide structures with terminal α - or β - linked N-acetylglucosamine, and to a lesser extent, bind to galactose residues

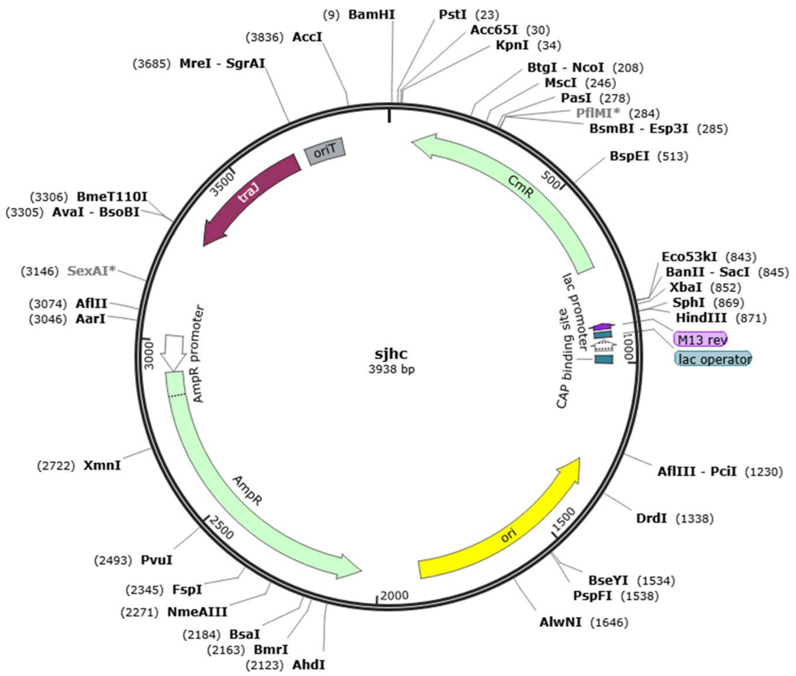

Supplementary Figure S1 Map of the plasmid Sjhc

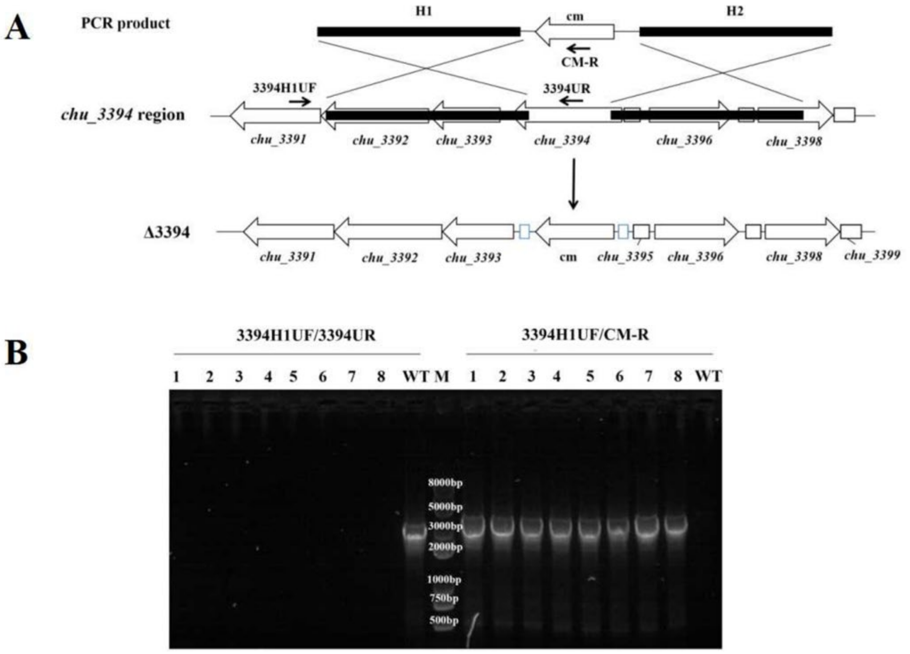

Supplementary Figure S2 The deletion of *chu_3394*. **(A)** Illustration of the deletion processes. Black arrows show approximate locations and orientations of primers; black-filled boxes indicate homologous arms; open arrowheads show arrangements and orientations of genes; open boxes

indicate residual genes. **(B)** PCR verification of the deletion of *chu_3394*. WT, wild type; 1-8, eight transformants of the deletion mutant.

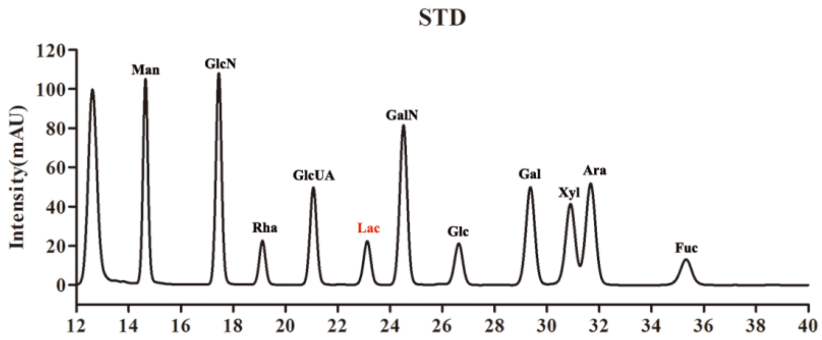

Supplementary Figure S3 HPLC chromatogram of standard monosaccharide PMP derivatization. Man—mannose; GlcN—glucosamine; Rha—rhamnose; GlcUA—glucuronic acid; Lac—lactose; GalN—galactosamine; Glc—glucose; Gal—galactose; Xyl—xylose; Ara—arabinose. Fuc—fucose. Lactose is used as an internal standard.

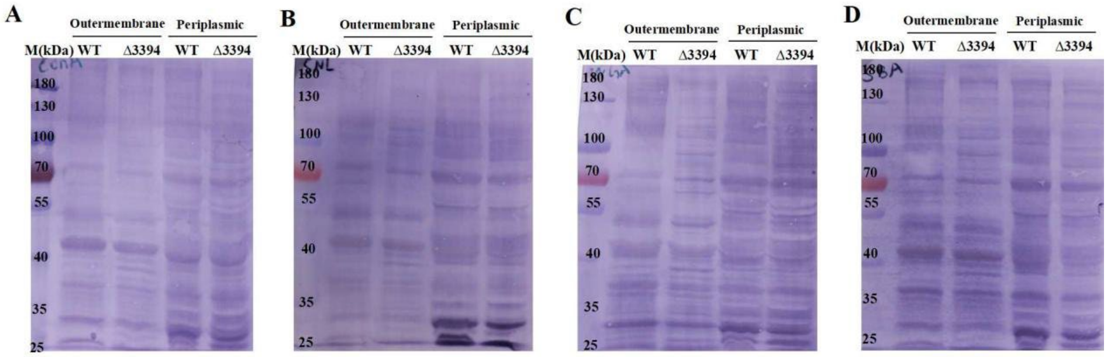

Supplementary Figure S4 The outermembrane and periplasmic proteins of wild-type and Δ3394 mutant were verified by western blotting and antibody-assisted lectin profiling. Concanavalin A **(A)**, Sambucus Nigra Lectin **(B)**, Wheat germ agglutinin **(C)** and Soybean Agglutinin **(D)** were used in this analysis.

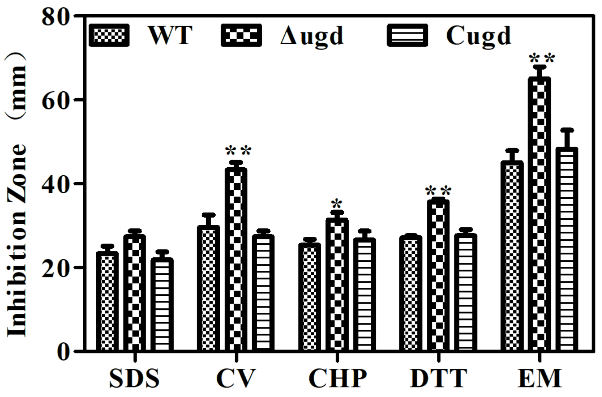

Supplementary Figure S5 The diameters of the inhibition zones for wild type, Δ*ugd*, and C*ugd* strains treated with various toxic agents. SDS—sodium dodecyl sulfate; CV—crystal violet; CHP—cumene hydroperoxide; DTT—dithiothreitol; EM—erythromycin. Significance reported as *p < 0.05, **p < 0.01.

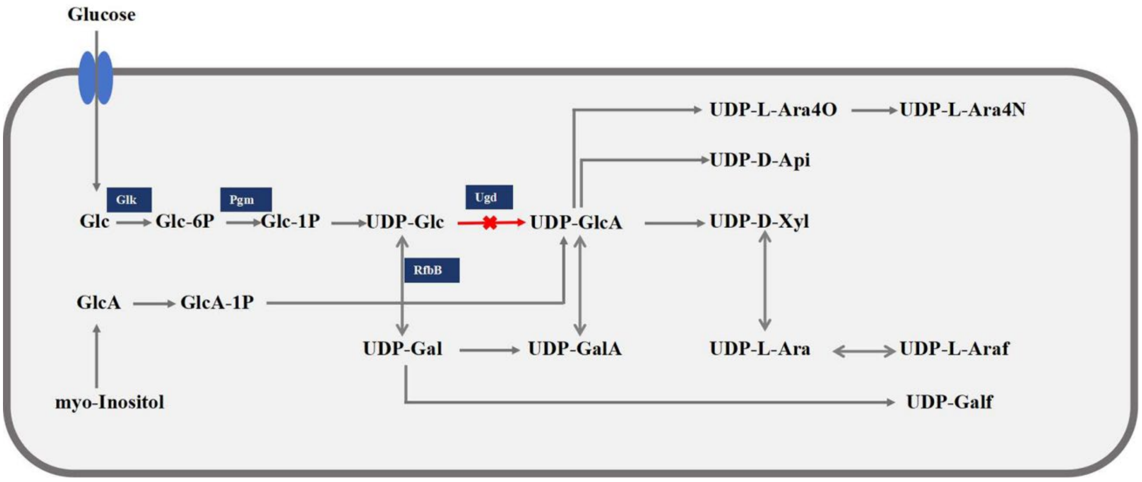

Supplementary Figure S6 Diagram illustrating Ugd's involvement in the nucleotide sugar biosynthesis pathways of *C. hutchinsonii*. The deletion of Ugd in *C. hutchinsonii* disrupts one of the UDP-GlcA biosynthesis pathways, which in turn affects the synthesis of various nucleotide sugars, including UDP-D-Xyl, UDP-D-Api, and UDP-L-Ara4O.
